# Indirect costs associated with deaths from the Ebola virus disease in West Africa

**DOI:** 10.1186/s40249-015-0079-4

**Published:** 2015-10-29

**Authors:** Joses Muthuri Kirigia, Felix Masiye, Doris Gatwiri Kirigia, Patricia Akweongo

**Affiliations:** Health Systems and Services Cluster, World Health Organization, Regional Office for Africa, Brazzaville, Congo; Department of Economics, University of Zambia, Lusaka, Zambia; The KEMRI-Wellcome Trust Research Programme, Nairobi, Kenya; School of Public Health, University of Ghana, Accra, Ghana

**Keywords:** Ebola virus disease, Non-health GDP loss, Indirect cost, Cost-of-illness

## Abstract

**Background:**

By 28 June 2015, there were a total of 11,234 deaths from the Ebola virus disease (EVD) in five West African countries (Guinea, Liberia, Mali, Nigeria and Sierra Leone). The objective of this study was to estimate the future productivity losses associated with EVD deaths in these West African countries, in order to encourage increased investments in national health systems.

**Methods:**

A cost-of-illness method was employed to calculate future non-health (NH) gross domestic product (GDP) (NHGDP) losses associated with EVD deaths. The future non-health GDP loss (NHGDPLoss) was discounted at 3 %. Separate analyses were done for three different age groups (< =14 years, 15–44 years and = >45 years) for the five countries (Guinea, Liberia, Mali, Nigeria, and Sierra Leone) affected by EVD. We also conducted a one-way sensitivity analysis at 5 and 10 % discount rates to gauge their impacts on expected NHGDPLoss.

**Results:**

The discounted value of future NHGDPLoss due to the 11,234 deaths associated with EVD was estimated to be Int$ (international dollars) 155,663,244. About 27.86 % of the loss would be borne by Guinea, 34.84 % by Liberia, 0.10 % by Mali, 0.24 % by Nigeria and 36.96 % by Sierra Leone. About 27.27 % of the loss is attributed to those aged under 14 years, 66.27 % to those aged 15–44 years and 6.46 % to those aged over 45 years. The average NHGDPLoss per EVD death was estimated to be Int$ 17,473 for Guinea, Int$ 11,283 for Liberia, Int$ 25,126 for Mali, Int$ 47,364 for Nigeria and Int$ 14,633 for Sierra Leone.

**Conclusion:**

In spite of alluded limitations, the estimates of human and economic losses reported in this paper, in addition to those projected by the World Bank, show that EVD imposes a significant economic burden on the affected West African countries. That heavy burden, coupled with human rights and global security concerns, underscores the urgent need for increased domestic and external investments to enable Guinea, Liberia and Sierra Leone (and other vulnerable African countries) to develop resilient health systems, including core capacities to detect, assess, notify, verify and report events, and to respond to public health risks and emergencies.

**Electronic supplementary material:**

The online version of this article (doi:10.1186/s40249-015-0079-4) contains supplementary material, which is available to authorized users.

## Multilingual abstract

Please see Additional file 1 for translations of the abstract into the six official working languages of the United Nations.

## Background

The estimated population of the six West African countries (Guinea, Liberia, Mali, Nigeria, Senegal and Sierra Leone) affected by the Ebola virus disease (EVD) was 225,181,000, representing 24.3 % of the entire African Region population. The annual population growth rate varied from 2.4 % in Guinea to 3.2 % in Liberia between 2003 to 2013. Between 36 and 49 % of the population lives in urban areas – a sizeable proportion resides in overcrowded and unsanitary informal settlements. The literacy rate among adults aged 15 years and above varied from 33 % in Mali to 61 % in Liberia. The gross national income per capita (GDPPC) varied between Int$ (international dollars) 790 in Liberia to Int$ 5360 in Nigeria. The percentage of population living below Int$ 1 per day varied from 34.1 % in Senegal to 83.8 % in Liberia [1].

Physicians, nursing and midwifery personnel, dentistry personnel, pharmaceutical personnel and psychiatrists are the key cadres of the health system. Except for in Nigeria – where the densities of physicians and nursing and midwifery personnel are higher than the African Region averages – the workforce densities were much lower than both the regional and global averages in the other five West African countries even before the EVD outbreak. The density of hospitals is also lower in EVD-affected countries compared to the regional and global averages. In addition, only Nigeria was spending 18 % of the total government budget on the health sector. This implies that even before the EVD outbreak occurred the other five countries did not meet the Abuja target of allocating at least 15 % of the national budget to the health sector. The per capita total expenditure on health (PCTEH), which was between Int$ 56 and Int$ 184 in EVD-affected countries, was lower than the African Region (Int$ 208) and global (Int$ 1173) averages [1]. Partially, as a result of years of civil conflict and underinvestment in health systems, the health services coverage even prior to the EVD outbreak was sub-optimal at best and disease surveillance capacities were weak especially in Guinea, Liberia and Sierra Leone [2].

Due to relatively weak national health systems, the EVD outbreak that was first reported in March 2014 in Guinea rapidly spread to other West African countries. As of 28 June 2015, a total of 27,543 cases of EVD were reported, of which 13.539 % were in Guinea, 38.725 % in Liberia, 0.029 % in Mali, 0.0726 % in Nigeria, 0.0034 % in Senegal and 47.631 % in Sierra Leone. The cumulative number of EVD-related deaths was 11,234, with 22.094 % of these in Guinea, 42.781 % in Liberia, 0.054 % in Mali, 0.071 % in Nigeria and 35 % in Sierra Leone [3].

According to the World Bank, apart from the loss of human lives and suffering, the EVD epidemic has also had a significant impact on the economies of the affected countries in terms of ‘…forgone output; higher fiscal deficits; rising prices; lower real household incomes and greater poverty’ [4]. The World Bank projected that the combined gross domestic product (GDP) loss for Guinea, Liberia and Sierra Leone, the three countries most severely affected by EVD, would be US$ 359 million in the short term (2014), US$ 129 million in a low Ebola prevalence scenario in the medium term (2015), and US$ 815 million in a high Ebola prevalence scenario in the medium term (2015) [4]. However, the World Bank analysis did not take into account the long-term impacts of mortality, failure to treat other health conditions as patients avoided health facilities in fear of contracting EVD, weakened healthcare supply capacity due to death of over 500 health workers, school closures and dropouts, and other shocks to livelihoods [4].

This paper attempts to answer the question: What is the impact of deaths associated with EVD on future non-health GDP (NHGDP) (i.e., GDP minus the total health expenditure) in the West African Region? The specific objective was to estimate future productivity losses of deaths associated with EVD in the West African Region in an effort to complement the World Bank projections and advocate for increased investments in health systems (including disease surveillance).

## Methods

### Conceptual framework

Fear, panic, morbidity and deaths associated with EVD had a negative impact on the economies of affected countries, especially Guinea, Liberia and Sierra Leone. This included the effects on real economic activities such as production, consumption, saving, investment, imports and exports. Changes in real economic activities, for example, changes in business cycles or in economic growth due to EVD would consequently affect future GDP. The GDP is the sum of households’ consumption expenditures (C), investment (I), government expenditures (G) and net exports (X), i.e., exports minus imports [5].

The category of ‘C’ comprises expenditures on consumable goods and services, such as health, education, food, clothing, transport, books, stationery, cooking utensils, entertainment, toiletries, newspapers, bicycles and motorcycles, private vehicles, etc. ‘C’ depends on real disposable income (i.e., income after taxation), wealth, the average price level, expectations, habits, demographic factors, etc.

The category ‘I’ denotes the purchase of capital goods or real assets on which a return is expected from the sales of production, e.g., buildings, public vehicles, tractors and machinery. ‘I’ depends on interest rates, expectations, business confidence and regulations. Usually money for investment comes from saved disposable income.

The category ‘G’ denotes purchase of goods and services by the government, e.g., roads, security forces (police and armed forces), medicines and supplies for public health facilities, stationary and salaries of civil servants. ‘G’ depends on total tax revenues (which depend on incomes) and borrowing.

The category ‘X’ refers to net exports, i.e., exports minus imports. Exports denote spending by foreigners on goods (e.g., minerals, agricultural products) and services (e.g., tourism and expatriate technical services) produced in EVD affected countries. The factors that determine exports include prices of locally produced goods relative to prices of imported goods (terms of trade), the exchange rate between local and foreign currencies, total income in foreign countries, trade policy in foreign countries (import taxes, tariffs, quotas, etc.) and trade sanctions or boycotts or bans. For example, some foreign countries banned exports of goods and services from EVD affected countries.

Imports refer to purchase of foreign consumer (e.g., clothes, petroleum and pharmaceuticals) and capital goods (e.g., Machinery, equipment) by citizens of EVD affected countries households, business enterprises, governments and parastatal institutions. Import expenditures are dependent on total income in EVD affected countries, prices of imported goods relative to the prices of locally produced goods, exchange rate, trade policy (import taxes, tariffs, quotas, etc.) and trade sanctions or boycotts or bans. For instance, some countries banned their airlines from flying to EVD affected countries and recalled non-medical expatriates hitherto working in those countries.

In EVD-affected countries, large numbers of people living off the agricultural sector fell seriously ill, some died and others fled their farms due to fear of contracting EVD. This in turn lead to a reduction in output; some mining, manufacturing and service industries closed down, and the volume of exports also diminished with the ban of commodities from EVD-affected countries. The decrease in economic activity and the ensuing increase in unemployment led to a decrease in disposable income and, hence, consumption, tax revenues, savings and investments [4].

This study employs a cost-of-illness method to estimate the impact of deaths associated with EVD on non-health components of future GDP [6, 7]. The ‘quantity of interest’ is the impact of deaths associated with EVD on the non-health components of GDP, because the use of health services or goods does not generate utility or welfare per se [8]. According to the economic theory of health production, consumers do not derive satisfaction from the consumption of medical services per se. For example, injections and radiology and surgical procedures are painful and some medicines are tasteless or have bitter taste. Thus, people in need use them not because of the satisfaction they provide but due to expected satisfaction from restored or improved health. The World Health Organization (WHO) [6] and Chisholm et al. [7] stipulate that the quantity of interest cannot be GDP, as medical care and health expenses actually form part of GDP. Instead, the impact of disease or injury on the non-health components of GDP is a more appropriate measurement of the quantity of interest.

### Calculations of the non-health GDP loss for each age group

The non-health GDP loss (NHGDPLoss) for each of the five EVD-affected countries was estimated separately for three different age groups: 0–14 years, 15–44 years and 45 years and above. The WHO EVD situation document reports numbers of cases and deaths classified in these same age categories [3].

The NHGDPLoss due to deaths associated with EVD for the 0–14 years age group in j^th^ country (*NHGDPLoss*_0 − 14_) is the product of the total number of discounted years of life lost (DYLL) above the minimum employment age lost, the per capita non-health GDP (NHGDPPC) in purchasing power parity (PPP) (*NHGDPPC*_*Int$*_) and the total number of deaths associated with EVD (TEVDD) in that age group (*TEVDD*_0 − 14_). The number of years of life lost (YLL) for this age group was calculated by subtracting the average age at death (AAD) (*AAD*_0 − 14_) from each country’s life expectancy at birth (LE). *AAD*_0 − 14_ is equal to 0 plus 14 years divided by 2, (0 + 14/2) = 7. A simple average was used, as the WHO EVD situation report, dated 1 July 2015, didn’t show the distribution of deaths by age. Because the legal minimum age of employment is 15 years, as according to article 2 of the International Labour Organization convention [9], the number of the future productive years of life lost (PYLL) equals each country’s average LE minus 14 years. For example, the PYLL among 0–14 year olds in Guinea is 44 years, i.e., LE of 58 years minus the sum of *AAD*_45 − *LE*_ (i.e., seven years) and years remaining to attain the minimum age of employment (i.e., seven years).

$$ PYL{L}_{0-14}=58-\Big[\left(0+14/2\right)+(7)=44\; years. $$

The formula for the 0–14 years age group is expressed as follows:

1 $$ \begin{array}{l} NHGDPLos{s}_{0-14}={\displaystyle \sum_{t=1}^n\left\{\left[1/{\left(1+r\right)}^t\right]\right.}\times \left[ NHGDPP{C}_{Int\$}\right]\times \left.\left[ TEVD{D}_{0-14}\right]\right\}=\\ {}\kern2.16em \left\{\left[1/{\left(1+r\right)}^1\right]\right.\times \left[ NHGDPP{C}_{Int\$}\right]\times \left.\left[ TEVD{D}_{0-14}\right]\right\}+\\ {}\kern2.16em \left\{\left[1/{\left(1+r\right)}^2\right]\right.\times \left[ NHGDPP{C}_{Int\$}\right]\times \left.\left[ TEVD{D}_{0-14}\right]\right\}+\dots +\\ {}\kern2.16em \left\{\left[1/{\left(1+r\right)}^n\right]\right.\times \left[ NHGDPP{C}_{Int\$}\right]\times \left.\left[ TEVD{D}_{0-14}\right]\right\}\end{array} $$

The NHGDPLoss due to deaths associated with EVD for the 15–44 years age group in j^th^ country (*NHGDPLoss*_15 − 44_) is the product of the total number of DYLL, NHGDPPC in PPP (*NHGDPPC*_*Int$*_) and the TEVDD in that age group (*TEVDD*_15 − 44_). The number of YLL for this age group was calculated by subtracting the AAD (*AAD*_15 − 44_) from each country’s average LE. *AAD*_15 − 44_ is equal to 15 plus 44 years divided by 2, (15 + 44/2). The formula for the 15–45 years age group is as follows:

2 $$ \begin{array}{l} NHGDPLos{s}_{15-44}={\displaystyle \sum_{t=1}^n\left\{\left[1/{\left(1+r\right)}^t\right]\right.}\times \left[ NHGDPP{C}_{Int\$}\right]\times \left.\left[ TEVD{D}_{15-44}\right]\right\}=\\ {}\kern2.16em \left\{\left[1/{\left(1+r\right)}^1\right]\right.\times \left[ NHGDPP{C}_{Int\$}\right]\times \left.\left[ TEVD{D}_{15-44}\right]\right\}+\\ {}\kern2.16em \left\{\left[1/{\left(1+r\right)}^2\right]\right.\times \left[ NHGDPP{C}_{Int\$}\right]\times \left.\left[ TEVD{D}_{15-44}\right]\right\}+\dots +\\ {}\kern2.16em \left\{\left[1/{\left(1+r\right)}^n\right]\right.\times \left[ NHGDPP{C}_{Int\$}\right]\times \left.\left[ TEVD{D}_{15-44}\right]\right\}\;\end{array} $$

Similarly, the NHGDPLoss due to deaths associated with EVD for those aged between 45 years and the age of LE in j^th^ country (*NHGDPLoss*_45 − *LE*_) is the product of the total number of DYLL, NHGDPPC in PPP (*NHGDPPC*_*Int$*_) and TEVDD in that age group (*TEVDD*_45 − *LE*_). The number of YLL for this age group was calculated by subtracting the AAD (*AAD*_45 − *LE*_) from each country’s average LE. *AAD*_45 − *LE*_ is equal to 45 plus LE divided by 2, e.g., as LE of Guinea was 58 years, *AAD*_45 − *LE*_ = (45 + 58/2) = 51.5. The formula for the 45 years and above age group is as follows:

3 $$ \begin{array}{l} NHGDPLos{s}_{=>45}={\displaystyle \sum_{t=1}^n\left\{\left[1/{\left(1+r\right)}^t\right]\right.}\times \left[ NHGDPP{C}_{Int\$}\right]\times \left.\left[ TEVD{D}_{=>45}\right]\right\}=\\ {}\kern2.16em \left\{\left[1/{\left(1+r\right)}^1\right]\right.\times \left[ NHGDPP{C}_{Int\$}\right]\times \left.\left[ TEVD{D}_{=>45}\right]\right\}+\\ {}\kern2.16em \left\{\left[1/{\left(1+r\right)}^2\right]\right.\times \left[ NHGDPP{C}_{Int\$}\right]\times \left.\left[ TEVD{D}_{=>45}\right]\right\}+\dots +\\ {}\kern2.16em \left\{\left[1/{\left(1+r\right)}^n\right]\right.\times \left[ NHGDPP{C}_{Int\$}\right]\times \left.\left[ TEVD{D}_{=>45}\right]\right\}\end{array} $$

In all three equations, 1/(1 + *r*)^*t*^ is the discount factor, *r* is the rate of discount of future losses, *t* is the first year of life lost and *n* is the final year of the total number of YLL per death associated with EVD. Thus, *NHGDPPC*_*Int$*_ is obtained by subtracting the per capita total health expenditure (PCTHE) from the per capita GDP (Int$ GDPPC) for each EVD-affected country. We used 2013 as the year on the basis of which losses potentially occurring in future years were discounted. The size of the discount factor decreases as one moves from the base year into future years, and, as a result losses in successive years are given a lower value relative to the same losses in the first year. This is due to existence of time preference – meaning individually and as a society we prefer to have dollars or resources now as opposed to later because we can benefit from them in the interim. Therefore, as Drummond et al. further explains, future dollar cost and benefit streams are reduced or ‘discounted’ to reflect the fact that dollars spent or saved in the future should not weigh as heavily in programme decisions as dollars spent or saved today [10]. The weight (discount factor) applied to the GDP losses of different years then depends not just on the discount rate, *r*, but also on the number of years, *t*, over which the discounting has been applied. When discounting is applied so that all GDP losses are re-valued relative to year 0 (i.e., 2013 in this study), the re-valued values are called present values [10].

### Calculation of losses in the total non-health GDP

The example below showing the calculation of loss associated with deaths from EVD in non-health GDP uses actual information on Guinea:

- TEVDD as of 28 June 2015 = 2482
- LE = 58 years
- Proportion of total deaths that occurred in the 0–14 years age group = 0.16
- Proportion of total deaths that occurred in the 15–44 years age group = 0.58
- Proportion of total deaths that occurred in the 45–58 years age group = 0.26
- TEVDD in the 0–14 years age group as of 28 June 2015 (*TEVDD*_0 − 14_) = (a) × (c) = 392
- TEVDD in the 15–44 years age group as of 28 June 2015 (*TEVDD*_15 − 44_) = (a) × (d) = 1438
- TEVDD in the 45–58 years age group as of 28 June 2015 (*TEVDD*_45 − 58_) = (a) × (e) = 652
- AAD in the 0–14 years age group (*AAD*_0 − 14_) = (0 + 14/2) = 7 years
- Years remaining to attain minimum employment age = 14 – 7 = 7 years
- Undiscounted PYLL in the 0–14 years age group = LE – (*AAD*_0 − 14_ +7) = 58 – (7 + 7) = 44 years
- Discounted PYLL in the 0–14 years age group = 24.25427392 years
- AAD in the 15–44 years age group (*AAD*_15 − 44_) = (15 + 44/2) = 29.5 years
- Undiscounted PYLL in the 15–44 years age group = LE – (*AAD*_15 − 44_) = 58 – 29.5 = 28.5 years
- Discounted PYLL in the 15–44 years age group = 19.18845459
- AAD in the 45–58 years age group (*AAD*_45 − 58_) = (45 + 58/2) = 51.5 years
- Undiscounted PYLL in the 45–58 years age group = LE – (*AAD*_15 − 44_) = 58 – 51.5 = 6.5 years
- Discounted PYLL in the 45–58 years age group = 6.230282955
- Int$ GDPPC = Int$ 1129.352
- PCTHE = Int$ 75.836
- NHGDPPC = GDPPC (Int$ 1129.352) − PCTHE (Int$ 75.836) = Int$ 1053.516
- *r* = 3 %
- (*NHGDPLoss*_0 − 14_) = (L)x(u)x(f) = 24.25427392 × 1053.516 × 392 = Int$ 10,016,488.13
- (*NHGDPLoss*_15 − 44_) = (o)x(u)x(g) = 19.18845459 × 1053.516 × 1438 = Int$ 29,069,665
- (*NHGDPLoss*_45 − 58_) = (r)x(u)x(h) = 6.230282955 × 1053.516 × 652 = Int$ 4,279,534.21
- Total NHGDPLoss in Guinea = (w) + (x) + (y) = Int$ 10,016,488 + Int$ 29,069,665 + Int$ 4,279,534 = Int$ 43,365,687

The above formulas were calculated using Excel to avoid errors. The NHGDPLoss for the other four West African countries were estimated the same way.

### Data sources and analysis

The data on LE, total child deaths, the GDPPC in PPP and PCTHE for each of the six EVD-affected West African countries were obtained from the World Health Statistics 2015 [1].

### Ethical clearance

This study was entirely based on analysis of published secondary data available in the public domain. Therefore, given that no human subjects were involved, the study did not require ethical clearance from the Ethics Review Committee of the WHO Regional Office for Africa.

## Results

**Fig. 1:**
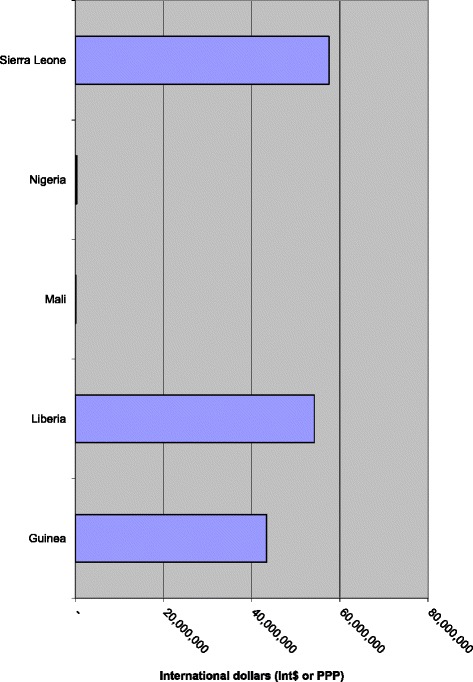
Discounted NHGDPLoss from deaths associated with EVD in the five West African countries (in Int$, PPP)

**Fig. 2:**
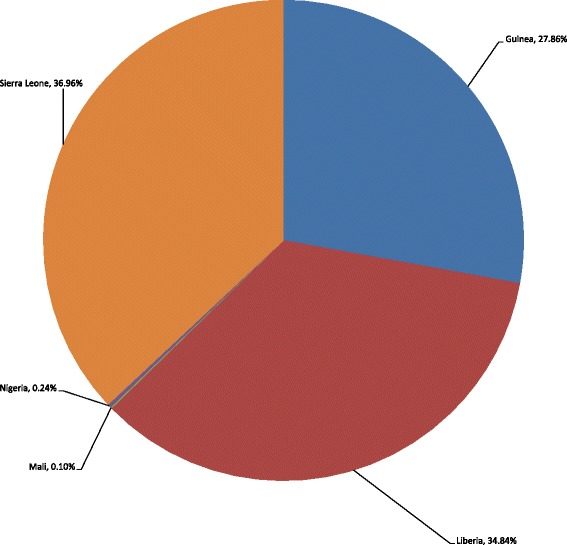
Percentage distribution of NHGDPLoss in the five West African countries

The 11,234 EVD deaths that occurred in the African Region as of 28 June 2015 could potentially decrease future total NHGDP of the affected countries by Int$ 155,663,244 (see Fig. 1). Approximately 36.96 % of the loss would be borne by Sierra Leone, 34.84 % by Liberia, 27.86 % by Guinea, 0.24 % by Nigeria and 0.10 % by Mali (see Fig. 2).

In terms of age, 27.27 % of the total NHGDPLoss was attributed to deaths that occurred in those aged 14 years or below, 66.27 % in those aged 15–44 years and 6.46 % in those aged over 45 years (see Table 1).

**Table 1:** Ebola death-related total discounted NHGDPLoss by age group (at 2013 prices)

Country	NHGDP loss (INT$) by age group			
	0–14 years	15–44 years	= >45 years	All ages
Guinea	10,011,713	29,078,934	4,277,754	43,368,401 (27.86 %)
Liberia	12,312,879	37,158,983	4,754,709	54,226,571 (34.84 %)
Mali	150,753	–	–	150,753 (0.10 %)
Nigeria	–	378,909	–	378,909 (0.24 %)
Sierra Leone	19,975,269	36,538,652	1,024,689	57,538,610 (36.96 %)
Total	42,450,614 (27.27 %)	103,155,478 (66.27 %)	10,057,152 (6.46 %)	155,663,244 (100 %)

The average NHGDP lost per death associated with EVD was Int$ 17,473 for Guinea, Int$ 11,283 for Liberia, Int$ 25,126 for Mali, Int$ 47,364 for Nigeria and Int$ 14,633 for Sierra Leone. The average NHGDPLoss per death associated with EVD in Nigeria was more than three times the overall average loss of Int$ 13,856 (see Table 2). These values were obtained by dividing a country’s total productivity loss by its TEVDD. The overall average NHGDPLoss per death of Int$ 13,856 was obtained by dividing all countries total productivity loss (Int$ 155,663,244) by total number of EVD deaths (11,234).

**Table 2:** Average NHGDPLoss per death associated with EVD (Int$)

Country	Total number of EVD deaths (A)	Total productivity loss in Int$, PPP (B)	Productivity loss per death associated with EVD in Int$, (C = B/A)
Guinea	2482	43,368,401	17,473
Liberia	4806	54,226,571	11,283
Mali	6	150,753	25,126
Nigeria	8	378,909	47,364
Sierra Leone	3932	57,538,610	14,633
Total	11,234	155,663,244	13,856^a^

^a^The overall average loss is NHGDPLoss (B) divided total deaths (A), i.e., $155,663,244/11234 = $13,856.44

The expected PYLL per death is dependent on the GDPPC. For instance, even though there were only eight deaths associated with EVD in Nigeria (a lower middle-income country), the NHGDPLoss per death associated with EVD was relatively high at Int$ 47,364. Low-income countries such as Guinea, Liberia and Sierra Leone with relatively high TEVDD of 2482, 4806 and 3932 have comparatively low productivity losses per death associated with EVD of Int$ 17,473, Int$ 11,283 and Int$ 14,633, respectively.

### Average GDP losses

The average NHGDPLoss per person for the five countries is shown in Table 3. The average NHGDPLoss per person for each country was estimated by dividing the country’s total NHGDPLoss by its population. Understandably, the average NHGDPLoss per person in Liberia and Sierra Leone is around three times that of Guinea due to the significantly smaller populations in Liberia and Sierra Leone. The NHGDPLoss per person in Mali and Nigeria was at least 300 % lower than those in Guinea, Liberia and Sierra Leone due the small number of deaths associated with EVD in Mali and Nigeria. The NHGDPLoss varied from a minimum of Int$ 0.0022 per person in Nigeria to a maximum of Int$ 12.63 per person in Liberia. Therefore, the average NHGDPLoss per person is largely dependent on both the population size and the number of deaths associated with EVD.

**Table 3:** Average NHGDPLoss per person (Int$)

Country	Population in 2013 (A)	Total productivity loss in Int$ (B)	Productivity loss per person in Int$, (C = B / A)
Guinea	11,745,000	43,368,401	3.69
Liberia	4,294,000	54,226,571	12.63
Mali	15,302,000	150,753	0.01
Nigeria	173,615,000	378,909	0.00
Sierra Leone	6,092,000	57,538,610	9.44
Total	211,048,000	155,663,244	0.74

### Sensitivity analysis

We applied a discount rate of 3 % to convert future loses into their present values, as this rate had been previously used in various arenas, including the WHO health systems’ performance assessment [11], global burden of disease studies [12], the Institute for Health Metrics and Evaluation’s global burden of disease studies [13] and health interventions economic evaluation studies in Africa [14]. However, to test the effect of the discount rate on the total expected NHGDPLoss estimate, a one-way sensitivity analysis was conducted at 5 % and 10 % discount rates. The 5 % discount rate reduced the total expected NHGDPLoss by Int$ 31.5 million (22 %) and the average non-health cost per death associated with EVD by Int$ 2802. The 10 % discount rate reduced the total NHGDPLoss by Int$ 75.8 million (48.7 %) and the average non-health cost per death associated with EVD by Int$ 6745. This indicates that the range of the NHGDPLoss is partially dependent on the discount rate used.

## Discussion

It is expected that the five West African countries will sustain a loss of about 0.03 % of their non-health GDP due to future YYL, as a result of 11,234 EVD-related deaths. Majority (66.27 %) of the total NHGDPLoss was attributed to deaths that occurred in those aged 15–44 years, i.e., the most productive bracket. And about a quarter of the NHGDPLoss was attributed to deaths that occurred in those aged 14 years or below, which may imply intergenerational economic effects of the EVD public health emergency.

The reader will recall that the World Bank projected that the combined GDP loss for Guinea, Liberia and Sierra Leone, the three countries most severely affected by EVD, would be US$ 359 million in the short term (2014), US$ 129 million in a low Ebola prevalence scenario in the medium term (2015), and US$ 815 million in a high Ebola prevalence scenario in the medium term (2015) [4]. Our study estimates that in addition to these short-term and medium term losses, the three countries are expected incur a loss of approximately Int$ 155.7 million due to premature EVD-related mortality. This loss is significant when one takes into account that nearly 40.9, 83.8 and 56.6 % of the populations in Guinea, Liberia and Sierra Leone live below the international poverty line of Int$1 per day [1].

In order to prevent the recurrence of the human and economic losses reported in this paper, in addition to those projected by the World Bank, there is an urgent need for increased investments in national health systems [15–18] – as well as in systems for addressing other determinants of health (such as education, shelter, clean sanitation and water) – as the three countries most severely affected by EVD (Guinea, Liberia and Sierra Leone) aim to meet their post-2015 Sustainable Development Goals.

In addition, the EVD outbreak in West Africa underscores the urgency of mobilizing domestic (public and private sector) and external resources to support full implementation of the International Health Regulations (2005) (IHR), which obliges for all States Parties to establish at local, intermediate and national public health response levels core capacities to detect, assess, notify, verify and report events, and to respond to public health risks and emergencies [19]. The WHO Review Committee on IHR partially attributed catastrophic effects of the EVD outbreak in the severely affected West African to the lack of core IHR capacities due to insufficient authority of national IHR focal point; the misconception that implementation of the IHR is the sole responsibility of ministries of health; dearth of involvement of sectors other than human health (e.g., animal health sectors); limited investment of national financial and human resources; high staff turnover; still recovering from devastating effects of recent civil conflict; and limited international solidarity to support such countries in developing, strengthening and maintaining core IHR capacities [20].

The three most affected countries (Guinea, Liberia and Sierra Leone) have developed health sector recovery plans, which, if fully funded and efficiently implemented, are expected to result in resilient health systems characterised by safe and healthy work settings; increased primary healthcare facility coverage in all areas; increased skilled workforce; increased community trust, ownership and access to essential health services; strengthened epidemic preparedness, surveillance and response compliant with international health regulations; improved health system governance; efficient intersectoral action on social determinants of health; and effective cross-border collaboration [21–23]. Following the July 2015 United Nations organized International Ebola Recovery Conference, the international community pledged more than five billion dollars to Guinea, Liberia and Sierra Leone to implement their recovery plans [24]. Therefore, there is optimism that if all the pledges are honoured the three countries would be able implement their recovery plans, and hence, build resilient health systems and IHR core capacities at all public health response levels.

### Limitations of the study

The present study had five main limitations. First, it does not include costs of alternative interventions that could prevent EVD-related morbidity and mortality and, thus, it cannot inform public health priority settings [25, 26]. Therefore, the purpose of this study was just to raise awareness among the public and policy-makers in the relevant ministries of health and finance about the negative impact of deaths associated with EVD on NHGDP.

Second, our study did not include direct healthcare costs such as those related to vaccines, drugs, tests, supplies, hospital, building and equipping EVD treatment centres, personnel involved in contact tracing and caring for patients, diagnostic and waste disposal equipment and physical facilities; direct non-healthcare costs of treatment such as transport; patients’ time costs; costs of volunteers’ time, or cost of family or friends accompanying patients to healthcare centres; loss in productivity due to morbidity before premature death; and intangible costs such as pain and grief due to premature loss of loved ones [27]. Thus, at best, this study is a partial cost-of-illness study.

Third, GDP calculations omit housework, including rearing children, preparing meals, caring for sick household members, and nursing and/or giving other assistance to the elderly. According to Becker, this undervalues the contribution of women, who are primarily responsible for household duties [28]. In addition, use of NHGDPPC, which is an average, disregards income distribution within a country and does not account for eternality (secondary negative effects) costs of production, e.g., environmental pollution from production processes.

Fourth, because EVD [29] put a massive strain on health systems, in terms of both direct (e.g., illness and death of the health workforce, abandonment of health facilities due to fear of contagion) and indirect effects, health systems were unable to properly accommodate patients suffering from other diseases (e.g., measles, HIV/AIDS, malaria, tuberculosis, maternal complications). The present study does not take into account the increase in morbidity and mortality from these other causes.

Lastly, the present study excludes the negative impact of EVD-related fear, panic and morbidity on exports, agriculture, industry (especially mining and tourism) and education due to closure of schools.

## Conclusions

In spite of alluded limitations, the estimates of human and economic losses reported in this paper, in addition to those projected by the World Bank, show that EVD imposes a significant economic burden on the affected West African countries. That heavy burden, coupled with human rights and global security concerns, underscores the urgent need for increased domestic and external investments to, in solidarity, support Guinea, Liberia and Sierra Leone (and other vulnerable African countries) to:

- Develop resilient local and national health systems [16];
- Develop core IHR capacities [19];
- Strengthen the interface between health and all other relevant sectors, including animal health, education, food, housing, transport, travel, tourism, water, sanitation, etc. [30];
- Strengthen the governance of national system of research for health to ensure production pertinent evidence and its use in the process of developing various systemic capacities [31]; and
- Strengthen political leadership and governance capacities at local, intermediate and national levels [32, 33].

In addition, there is urgent need for further research to determine:

- Economic feasibility of various systems of ensuring universal health coverage.
- Feasibility of community/village multi-sectoral development committees (agriculture, education, health, water and sanitation, transport, security); community/village health committees; and health facility management committees (with community representation).
- Current core IHR capacities, bottlenecks and economically feasible ways of developing and sustaining IHR capacities.
- Functionality of community and district health systems: management structures, managerial processes, policy to guide health sector reforms at district level, collaboration with other actors (e.g., other services providers); availability and use of guidelines, standards and norms at health facilities; supervision and monitoring; medicines management; referral mechanisms; health management information system; priority health activities; community involvement; health and health-related resources funding and financial management; human resources; infrastructure, equipment, supplies and logistics.
- Performance of national system of research for health and actions for improvement.
- Feasible approaches for changing negative health-related community behaviours, which makes them vulnerable to infectious diseases such as EVD.

## Additional file

Additional file 1:
**Multilingual abstracts in the six official working languages of the United Nations.** (PDF 356 kb)

## Abbreviations

AAD: Average age at death
AAD_0–14_: Average age at death for those aged 0–14 years
AAD_15–44_: Average age at death for those aged 15–44 years
AAD_=>45_: Average age at death for those aged 45 years and above
C: Consumption
DYLL: Discounted years of life lost
EVD: Ebola virus disease
GDP: Gross domestic product
GDPPC: Per capita GDP
I: Investment
Int$: International dollars
LE: Life expectancy at birth
*n*: Final year of the total number of years of life lost per death associated with EVD
*NHGDPC*_*Int$*_: Per capita non-health GDP in PPP
NHGDPLoss: Non-health GDP loss
NHGDPLoss_0–14_: Non-health GDP loss in the 0–14 years age group
NHGDPLoss_15–44_: Non-health GDP loss in the 15–44 years age group
NHGDPLoss_=>45_: Non-health GDP loss in the 45 years and above age group
PCTHE: Per capita total health expenditure
PPP: Purchasing power parity
PYLL: Productive years of life lost
*r*: Rate of discount of future losses
*t*: First year of life lost
TEVDD: Total number of deaths associated with EVD
TEVDD_0–14_: Total number of deaths associated with EVD in the 0–14 years age group
TEVDD_15–44_: Total number of deaths associated with EVD in the 15–44 years age group
TEVDD_45–45_: Total number of deaths associated with EVD in the = >45 years age group
WHO: World Health Organization
X: Net exports, i.e., exports minus imports
