# Supplementary material for: Indirect costs associated with deaths from the Ebola virus disease in West Africa
Source: Infect Dis Poverty. 2015 Oct 29;4:45. doi: 10.1186/s40249-015-0079-4 (PMC4625462; doi:10.1186/s40249-015-0079-4)
Supplement: Additional file 1: — Multilingual abstracts in the six official working languages of the United Nations. (PDF 356 kb) [file 40249_2015_79_MOESM1_ESM.pdf]

Translation of the abstract into the six official working languages of the United Nations

التكاليف غير المباشرة المرتبطة بالوفيات الناتجة عن مرض فيروس الإيبولا فى غرب أفريقيا

يوسى موثورى كيريجيا، فيليكس ماسايا، دوريس جاتويري كيريجيا وباتريشيا أكويونجو

الملخص

الخلفية: فى الثامن والعشرون من شهر يونيو من عام 2015 كان هناك إجمالى 11,234 حالة وفاة بسبب مرض فيروس الإيبولا (EVD) فى خمس بلدان فى غرب أفريقيا هى غينيا وليبيريا ومالى ونيجيريا وسيراليون، والهدف من هذه الدراسة هو تقدير الخسائر الانتاجية المستقبلية المرتبطة بحالات الوفاة بسبب مرض فيروس الإيبولا فى هذه البلاد فى غرب أفريقيا من أجل تشجيع زيادة الاستثمارات فى الأنظمة الصحية الوطنية.

الأساليب: تم إستخدام طريقة تكلفة المرض لحساب إجمالى الخسائر المستقبلية الغير الصحية فى الناتج المحلى والمرتبطة بحالات الوفاة بمرض فيروس الإيبولا، تم خصم الخسائر الغير صحية المستقبلية فى الناتج المحلى الإجمالى بنسبة 3% وتم عمل تحليلات منفصلة لثلاث فئات عمرية مختلفة هى أقل من أو يساوى 14 عام، من 14 الى 44 عام وأكبر من أو يساوي 45 عام وذلك فى البلدان الخمس سالفة الذكر المتضررة بمرض فيروس الإيبولا وقمنا أيضا بعمل تحليل الحساسية فى إتجاة واحد عند معدلات خصم 5 و 10% لقياس مدى تأثيرهما على إجمالى الخسارة المستقبلية الغير صحية المتوقعة فى الناتج المحلى.

النتائج: تم تقدير القيمة المخصومة من إجمالى الخسارة المستقبلية الغير صحية الناتجة عن 11,234 حالة وفاة بسبب مرض فيروس الإيبولا بـ 155,663,244 دولار أمريكى وكانت نسبة 27.86% من هذه الخسارة متوقعة من غينيا و نسبة 34.84% من ليبيريا و 0.1% من مالى و 0.24% من نيجيريا و 36.96% من سيراليون، ويعزى حوالى 27.27% من الخسارة إلى الذين تقل اعمارهم عن 14 عام ، 66.27% لأولئك الذين تتراوح اعمارهم بين 15-44 عاما، و6.46% لأولئك الذين تزيد اعمارهم عن 45 عاما، كما كان متوسط الخسارة المستقبلية الغير صحية لكل حالة وفاة بمرض فيروس الإيبولا تقدر بـ 17,473 دولار لغينيا و 11,283 دولار لليبيريا و 25,126 لمالى و 47,364 لنيجيريا و 14,633 لسيراليون.

الخلاصة: على الرغم من القيود الملمح إليها فإن تقديرات الخسائر البشرية والإقتصادية التى ذكرت فى هذه الدراسة بالإضافة إلى تلك المتوقعة من قبل البنك الدولى تظهر أن مرض فيروس الإيبولا يفرض عبئا إقتصاديا كبيرا على بلدان غرب أفريقيا المتضررة وهذا العبء الثقيل إلى جانب حقوق الإنسان والمخاوف الأمنية العالمية يؤكد على الحاجة الملحة لزيادة الإستثمارات المحلية والخارجية لتمكين غينيا وليبيريا وسيراليون، وغيرها من البلدان الأفريقية الضعيفة، من تطوير نظم صحية مرنة تشتمل على قدرات أساسية لكشف وتقييم والتحقق والإبلاغ عن الإصابات، وللإستجابة للمخاطر الصحية العامة والطوارئ.

Translated from English version into Arabic by Mohamed R. Habib, through

CHINESE CENTER FOR DISEASE CONTROL AND PREVENTION
NATIONAL INSTITUTE OF PARASITIC DISEASES

西非埃博拉病毒病死亡病例的相关间接费用

Joses Muthuri Kirigia, Felix Masiye, Doris Gatwiri Kirigia, Patricia Akweongo

摘要

引言：2015 年 6 月 28 日，西非五国（几内亚、利比里亚、马里、尼日利亚和塞拉利昂）已有 11,234 例埃博拉病毒病（EVD）死亡病例。本研究旨在评估西非五国 EVD 死亡病例造成的未来生产力损失，以鼓励国家卫生系统增加投资。

方法：采用疾病成本法计算与 EVD 死亡病例相关的非健康国内生产总值损失（(NHGDPLoss）。NHGDPLoss 折扣 3%。在受 EVD 影响的上述 5 个国家中对 3 个不同年龄患病人群（≤14 岁、15-44 岁和≥45 岁）进行独立分析。并对 5%和 10%的贴现率进行了单向灵敏度分析，以评估其对预期 NHGDPLoss 的影响。

结果：11,234 例 EVD 死亡病例造成的未来 NHGDPLoss 贴现值估计为 155,663,244 国际元（Int$）。其中，几内亚占 27.86%，利比里占 34.84%，马里占 0.10%，尼日利亚占 0.24%，塞拉利昂占 36.96%。14 岁以下患者造成的损失占 27.27%，15-44 岁占

66.27%，45 岁以上占 6.46%。几内亚每例 EVD 死亡病例的 NHGDPLoss 约为 Int$ 17，473，利比里亚：Int$ 11，28，马里：Int$ 25，126，尼日利亚：Int$ 47，364，塞拉利昂：Int$ 14，633。

结论：本研究报道的人类和经济损失的估值表明，除由世界银行资助的项目外，EVD 对相关西非国家已造成显著经济负担。加之人权和全球安全问题，这种沉重的负担强调几内亚、利比里亚和塞拉利昂（以及其他弱势非洲国家）急需增加国内外投资以制定弹性的卫生系统，包括提高检测、评估、通报、核实和报告事件等的核心能力，以及应对公共卫生风险和突发事件的能力。

Translated from English version into Chinese by Chen Jin, edited by Yang Pin, through

Coûts indirects associés aux décès dus à la fièvre d'Ébola en Afrique de l'Ouest

Joses Muthuri Kirigia, Felix Masiye, Doris Gatwiri Kirigia, Patricia Akweongo

Résumé

Contexte : Au 28 juin 2015, on dénombrait un total de 11 234 décès causés par la fièvre d'Ébola dans cinq pays d'Afrique de l'Ouest (Guinée, Liberia, Mali, Nigeria et Sierra Leone). Le but de la présente étude était d'estimer les pertes de productivité futures associées à cette mortalité dans ces pays, afin d'encourager des investissements accrus dans les systèmes de santé publique nationaux.

Méthodes : Une méthode de calcul du coût de la maladie a été employée pour évaluer les futures pertes de produit intérieur brut (PIB) non consacré à la santé (PIB-NS) résultant de la mortalité due au virus Ebola. Ces pertes ont été chiffrées à 3 %. Des analyses séparées ont été effectuées pour trois groupes d'âge différents (<=14 ans, 15–44 ans et =>45 ans) pour les cinq pays affectés par le virus Ebola (Guinée, Liberia, Mali, Nigeria et Sierra Leone). Nous avons également réalisé une analyse de sensibilité unidirectionnelle avec un taux de réduction de 5 % et 10 % afin d'évaluer l'impact de ces taux sur les pertes de PIB-NS attendues.

Résultats : La valeur réduite des futures pertes e PIB-NS résultant des 11 234 décès dus au virus Ebola a été estimée à 155 663 244 Int$ (dollars internationaux). Cette perte affecterait pour 27,86 % la Guinée, 34,84 % le Liberia, 0,10 % le Mali, 0,24 % le Nigeria et 36,96 % le Sierra Leone. Environ 27,27 % de la perte est attribuée au groupe des moins de 14 ans, 66,27% aux 15–44 ans et 6,46 % aux plus de 45 ans. La perte de PIB-NS moyenne par mort due au virus Ebola a été estimée à 17 473 Int$ pour la Guinée, 11 283 Int$ pour le Liberia, 25 126 Int$ pour le Mali, 47 364 Int$ pour le Nigeria et 14 633 pour le Sierra Leone.

Conclusion : Malgré les limites évoquées, les estimations des pertes humaines et économiques rapportées dans notre article, ajoutées aux projections de la Banque Mondiale, montrent que le virus Ebola pèse lourdement sur l'économie des pays d'Afrique de l'Ouest affectés. Ce poids, couplé aux problèmes de droits humains et de sécurité mondiale, accentue l'urgente nécessité d'accroître les investissements intérieurs et extérieurs afin de permettre à la Guinée, au Liberia et au Sierra Leone (comme d'ailleurs à d'autres pays africains vulnérables) de mettre en place des systèmes de santé publique résilients, dotés des capacités nécessaires pour détecter, évaluer, notifier, vérifier et rapporter les événements et pour répondre aux risques et urgences de santé publique.

Translated from English version into French by Suzanne Assenat, through

Непрямые затраты, связанные со смертями от болезни, вызванной вирусом Эбола в Западной Африке

Жозе Мутури Кириджа, Феликс Мазийе, Дорис Гатвири Кириджа, Патриция Аквеонго

Реферат

История вопроса: На 28 июня 2015 года в пяти западноафриканских странах (Гвинея, Либерия, Мали, Нигерия и Сьерра-Леоне) зарегистрировано 11234 смертей от болезни, вызванной вирусом Эбола (EVD). Целью данного исследования являлась оценка будуших потерь производительности в связи со смертями от болезни, вызванной вирусом Эбола, в этих западноафриканских странах, которая должна стимулировать увеличение капиталовложений в национальные системы здравоохранения.

Методы: Для расчета будущих потерь валового внутреннего продукта, не связанных со здоровьем населения, вызванных смертями от вируса Эбола, использовался метод оценки стоимости болезни. Будущие потери валового внутреннего продукта, не связанные со здоровьем населения, были оценены в размере 3%. Был проведен раздельный анализ для трех возрастных групп (<=14 годам, 15–44 лет и =>45 лет) в пяти странах (Гвинея, Либерия, Мали, Нигерия и Сьерра-Леоне), пораженных вирусом Эбола. Нами также проведен односторонний анализ при 5% и 10% от дисконтированной стоимости для оценки влияния на ожидаемые потери валового внутреннего продукта, не связанные со здоровьем населения.

Результаты: Дисконтированная стоимость потерь валового внутреннего продукта, не связанных со здоровьем населения, вследствие 11234 смертей от болезни, вызванной вирусом Эбола (EVD), была оценена в размере 155663244 международных долларов (Int$). Около 27.86% потерь понесет Гвинея, 34.84% - Либерия, 0.10% - Мали, 0.24% - Нигерия и 36.96% - Сьерра-Леоне. Около 27.27% потерь относится к населению моложе 14 лет, 66.27% к возрастной группе 15–44 лет и 6.46% к населению старше 45 лет. Средние потери валового внутреннего продукта, не связанные со здоровьем населения, вызванные смертями от вируса Эбола, оцениваются в размере 17473 международных долларов для Гвинеи, 11283 международных долларов для Либерии, 25126 международных долларов для Мали, 47364 международных долларов для Нигерии и 14633 международных долларов для Сьерра-Леоне.

Выводы: Несмотря на указанные ограничения, оценки человеческих и экономических потерь, приводимые в данном отчете, в дополнение к потерям, прогнозируемым Всемирным банком, показывают, что вирус Эбола возлагает значительное экономическое бремя на пораженные им западноафриканские страны. Это тяжелое бремя вкупе с озабоченностью по поводу вопросов прав человека и общей безопасности еще более усиливает безотлагательную необходимость увеличения внутренних и внешних капиталовложений, которые позволили бы Гвинее, Либерии, Сьерра-Леоне (и другим уязвимым африканским странам) развить устойчивые системы здравоохранения, обладающие основополагающими возможностями выявления, оценки, проверки и уведомления о рисках и экстренних ситуациях, связанных со здоровьем населения.

Translated from English version into Russian by Alena Hrybouskaya, through

TRANSLATORS
WITHOUT BORDERS

Costos indirectos asociados con las muertes por la enfermedad por el virus del ébola en África Occidental

Joses Muthuri Kirigia, Felix Masiye, Doris Gatwiri Kirigia, Patricia Akweongo

Resumen

Antecedentes: Al 28 de junio de 2014, hubo un total de 11234 muertes por la enfermedad por el virus del ébola (EVE) en cinco países de África Occidental (Guinea, Liberia, Mali, Nigeria y Sierra Leona). El objetivo de este estudio fue de estimar las futuras pérdidas de productividad asociadas con las muertes por EVE en estos países del África Occidental, con el objeto de fomentar una mayor inversión en los sistemas nacionales de salud.

Métodos: Se utilizó un método de costo-de-la-enfermedad para calcular las pérdidas futuras de producto bruto interno (PBI) no asociado a salud en relación con las muertes por EVE. Las futuras pérdidas de PBI no asociado a salud se descontaron a un 3%. Se realizaron análisis por separado para tres grupos diferentes de edades (<=14 años, 15–44 años y =>45 años) para los cinco países afectados por EVE (Guinea, Liberia, Mali, Nigeria, y Sierra Leona). También llevamos a cabo un análisis de sensibilidad unidireccional a tasas de descuento del 5 y el 10% para medir su impacto sobre las pérdidas previstas en el producto bruto interno no asociado a salud.

Resultados: El valor descontado para pérdidas futuras de PBI no asociado a salud debido a las 11234 muertes asociadas con EVE se estimó en $155.663.244 dólares internacionales. Aproximadamente 27,86% de la pérdida era atribuible a Guinea, 34,84% a Liberia, 0,10% a Mali, 0,24% a Nigeria y 36,96% a Sierra Leona. Cerca del 27,27% de la pérdida se atribuye a aquellas personas de menos de 14 años de edad, 66,27% a aquellas personas entre 15 y 44 años de edad y 6,46% a aquellas personas de más de 45 años. La pérdida promedio de PBI no asociado a salud por muerte por EVE se estimó en $17.473 dólares internacionales para Guinea, $11.283 dólares internacionales para Liberia, $25.126 dólares internacionales para Mali, $47.364 dólares internacionales para Nigeria y $14.633 dólares internacionales para Sierra Leona.

Conclusión: A pesar de las citadas limitaciones, las estimaciones de pérdidas humanas y económicas reportadas en este estudio, además de aquellas que proyecta el Banco Mundial, muestran que la EVE impone una carga económica significativa para los países afectados de África Occidental. La pesada carga, sumada a las preocupaciones sobre derechos humanos y seguridad global, pone énfasis en la necesidad de mayores inversiones domésticas e internacionales para permitir que Guinea, Liberia y Sierra Leona (y otros países vulnerables de África) puedan desarrollar sistemas de salud resistentes, incluyendo capacidades básicas para la detección, evaluación, notificación, verificación y reporte de eventos y para poder responder a los riesgos y emergencias de salud pública.

Translated from English version into Spanish by Maria Alejandra Aguada, through

TRANSLATORS WITHOUT BORDERS
